# Hb A1c Separation by High Performance Liquid Chromatography in Hemoglobinopathies

**DOI:** 10.1155/2016/2698362

**Published:** 2016-02-16

**Authors:** Vani Chandrashekar

**Affiliations:** Department of Hematology, Apollo Hospitals, No. 21, Off Greams Road, Greams Lane, Chennai 600006, India

## Abstract

Hb A1c measurement is subject to interference by hemoglobin traits and this is dependent on the method used for determination. In this paper we studied the difference between Hb A1c measured by HPLC in hemoglobin traits and normal chromatograms. We also studied the correlation of Hb A1c with age. Hemoglobin analysis was carried out by high performance liquid chromatography. Spearman's rank correlation was used to study correlation between A1c levels and age. Mann-Whitney *U* test was used to study the difference in Hb A1c between patients with normal hemoglobin and hemoglobin traits. A total of 431 patients were studied. There was positive correlation with age in patients with normal chromatograms only. No correlation was seen in Hb E trait or beta thalassemia trait. No significant difference in Hb A1c of patients with normal chromatograms and patients with hemoglobin traits was seen. There is no interference by abnormal hemoglobin in the detection of A1c by high performance liquid chromatography. This method cannot be used for detection of A1c in compound heterozygous and homozygous disorders.

## 1. Introduction

Hb A1c represents the fraction of hemoglobin bound to glucose [1]. Some of the methods for measuring A1c are boronate affinity, electrophoresis, cation exchange chromatography, and immunoassay [2]. Hemoglobinopathies can affect A1c measurement in various ways—by altering glycation of hemoglobin, by causing hemolysis thus reducing glycation time, and also by producing a peak in chromatograms rendering interpretation difficult [3]. The effect of hemoglobinopathies on glycated hemoglobin is method dependent [4]. It was seen that low or high value for Hb A1c was reported in the presence of hemoglobinopathies while using high performance liquid chromatography, immunoassay, and immunoagglutination methods [4]. It was seen that boronate affinity method showed acceptable values in the presence of hemoglobinopathies [4]. Discrepancies between home blood glucose monitoring and A1c measurements have been reported to be around 20.3% and these discrepancies have been seen in patients with hemoglobinopathies [5]. Presence of Hb S or Hb C has been reported to affect A1c measurements [2, 6–10]. In another study evaluating 14 methods for Hb A1c measurement, there was no interference by Hb C and Hb S on Hb A1c measurement by ion exchange chromatography [11]. With Hb D and Hb E traits certain ion exchange chromatography methods were found to be acceptable with no interference on A1c measurement [12].

In this study we studied the difference in levels of Hb A1c in patients with and without hemoglobinopathies. We also studied the chromatograms for possible interference by abnormal hemoglobin.

## 2. Materials and Methods

**Figure 1:**
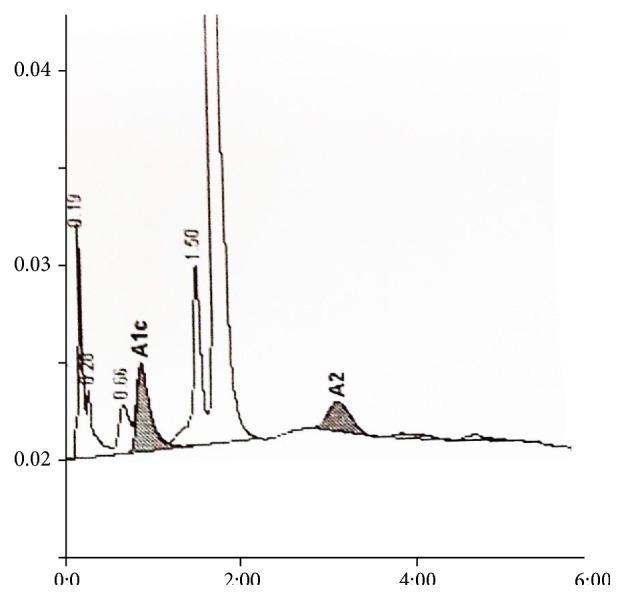
A1c of 4.7% at retention time of 0.87 minutes in a normal chromatogram.

**Figure 2:**
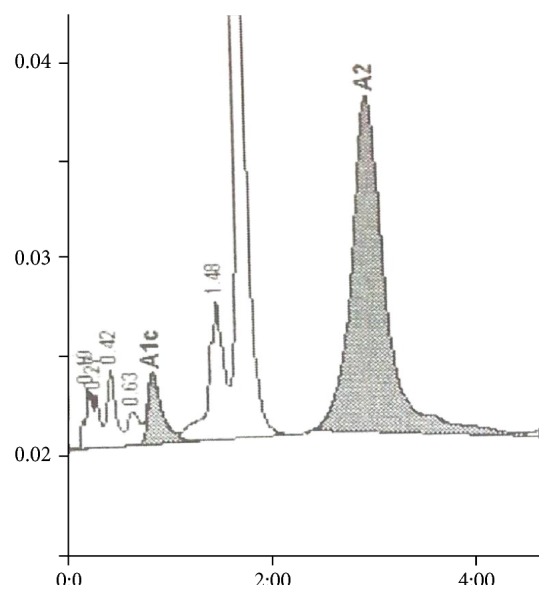
A1c of 4.8% at retention time of 0.83 minutes in Hb E trait.

EDTA anticoagulated blood samples were collected from patients after informed consent for complete blood counts and hemoglobin analysis. Patients with history of recent blood transfusion within the preceding three months were not included in the study. For complete blood counts samples were analyzed within four hours of collection in LH 780 analyzer (Beckman Coulter). High performance liquid exchange chromatography (HPLC) using Hemoglobin A1c/A2/F BIO-RAD D-10 dual program on D-10 analyzer (Bio-Rad) separated the hemoglobin fractions. The samples were directly loaded into the instrument after which they were automatically diluted and injected into the analytical cartridge. Then a programmed buffer gradient of increasing ionic strength was delivered to the cartridge. The hemoglobin separated based on its ionic interactions with the cartridge. The eluted hemoglobin flows through a flow cell where its absorbance at 415 nm was recorded. The D-10 software performs reduction of the raw data and a chromatogram is generated which will have the peaks in the following order: Hb A1a, Hb A1b, Hb F, LA1c/CHb-1, Hb A1c, Hb P3, Hb A0, and Hb A2. There are two levels of calibration for Hb F, Hb A1c, and Hb A2. Hb A1c is calculated after subtracting the labile and carbamylated portion. The chromatograms were analyzed for presence of Hb A1c peak and variant peaks (Figures 1 and 2). The variant hemoglobin was identified by its retention times and comparing it with manufacturer assigned retention times. If the total chromatogram area exceeded 4 million *μ*volts or was less than 1 million *μ*volts the results were considered inaccurate. Variant hemoglobin (Hb) D, Hb E, and Hb S separated into windows. Hb E trait separated as Hb A2 (30–39%) whereas Hb S and Hb D separated into a window beyond Hb A2. Beta thalassemia traits were identified by elevated Hb A2 (but less than 9%) and minimal or no elevation of Hb F. Beta thalassemia major was identified by high Hb F levels whereas homozygous E had Hb E levels beyond 85% and minimal elevation of Hb F. Hb A1c was expressed as percentage of the total hemoglobin. The A1c levels were used to subdivide the patients into normal and diabetic using the classification of the American Diabetes Association (less than 5.7% as normal and 6.5% and above as diabetes).

### 2.1. Statistical Methods

Data were entered into excel worksheets. Normality was determined by normal probability plots. Since Hb A1c showed a distribution which was not normal, nonparametric tests—Spearman's ranked correlation (Vassar stats) and Mann-Whitney *U* test—were used. Spearman's ranked correlation was used to study the correlation and level of significance between age and Hb A1c measurements in normal chromatograms as well as traits. Mann-Whitney *U* test (Vassar stats) was conducted to test the null hypothesis that Hb A1c levels between the two groups—normal hemoglobin and hemoglobin traits—are similar. Significant *p* level was considered to be less than 0.05.

### 2.2. Observations

A total of 431 chromatograms and complete blood counts were analyzed. Among these, 71 patients with diabetes (Hb A1c of 6.5 and more) and low Hb A1c (less than 4%) were excluded. From the remaining 360 patients, normal chromatogram was seen in 186. There were 122 females and 64 males. Median age was 32 years. Youngest patient was 17 years old whereas the oldest was 87 years old. Median hemoglobin was 105 gm/L. Hemoglobin varied from 33 to 162 gm/L. Hb A1c peaks were visualised in all chromatograms. Median Hb A1c was 5.3% with a range from 4 to 6.4%. Median Hb A was 85.8% with a range from 79.5 to 89.2%.

A total of 119 patients had hemoglobinopathy or beta thalassemia trait which included 63 with beta thalassemia trait, 48 with Hb E trait, five with Hb S trait, and three with Hb D trait. Patients with sickle cell trait and Hb D trait were excluded as they were few in number. Among patients with beta thalassemia trait, there were 36 males and 27 females. The median age was 32 years. Age varied from one to 70 years. Median hemoglobin was 109 gm/L. Hemoglobin varied from 74 to 149 gm/L. Hb A1c peaks were visualised in all chromatograms. Median Hb A1c was 5.4% with a range from 4.2 to 6.3%. Median Hb A was 82.4% with a range from 74.8 to 85.2%. Median Hb A2 was 5.3% with a range from 3.6 to 7%. Among patients with Hb E trait, there were 24 males and 24 females. The median age was 33.5 years. Age varied from one to 72 years. Median hemoglobin was 106 gm/L. Hemoglobin varied from 57 to 154 gm/L. Hb A1c peaks were visualised in all chromatograms. Median Hb A1c was 5.2% with a range from 4.4 to 6.2%. Median Hb A was 61.0% with a range from 56.8 to 69.0%. Median Hb E+ Hb A2 was 27.3% with a range from 20.4 to 30.2%.

Compound heterozygous/homozygous hemoglobinopathies were seen in 55 patients. Homozygous Hb E was seen in 37 patients, beta thalassemia major was seen in eight, compound heterozygous Hb E/beta thalassemia was seen in five, homozygous sickle was seen in three, Hb H was seen in one, and compound heterozygous Hb S/beta thalassemia was seen in one patient. There were 28 males and 27 females.

### 2.3. Spearman's Ranked Correlation

Age had a significant positive correlation with Hb A1c in normal individuals, *r*_*s*_ = 0.39, *p* < 0.000001 (df = 184, *n* = 186). No significant correlation was seen in beta thalassemia traits, *r*_*s*_ = 0.24, *p* = 0.05 (df = 61, *n* = 63). Age had no significant relationship with Hb A1c in Hb E trait, *r*_*s*_ = 0.06, *p* = 0.6 (df = 46, *n* = 48).

### 2.4. Mann-Whitney *U* Test

Hb A1c from normal patients does not differ significantly from patients with traits, *z* = −0.9, *p* = 0.368. Patients with normal chromatograms had a rank of 145.5 whereas patients with traits had a rank of 154.8.

Hb A1c from normal patients does not differ significantly from patients with Hb E trait, *z* = 0.6, *p* = 0.54. Patients with normal hemoglobin had a rank of 118.9 whereas patients with Hb E trait had a rank of 112.3. Hb A1c from normal patients does not differ significantly from patients with beta thalassemia trait, *z* = −1.82, *p* = 0.06. Patients with normal hemoglobin had a rank of 120.2 whereas patients with beta thalassemia trait had a rank of 139.3.

Differences between patients with normal hemoglobin and traits are tabulated in Table 1.

**Figure 3:**
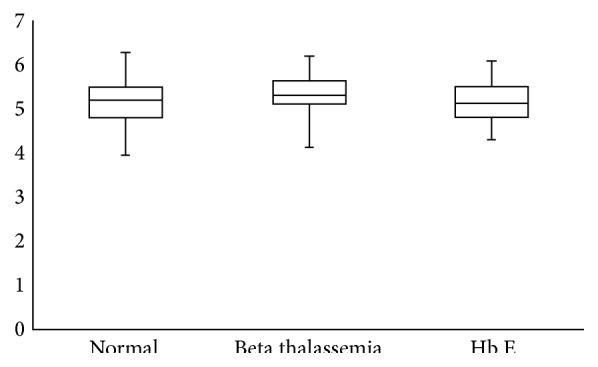
Box plot showing quartiles 1, 2, and 3 for Hb A1c in normal chromatograms, beta thalassemia trait, and Hb E trait.

Figure 3 is a box plot summary of Hb A1c distribution. Note that the medians are 5.3, 5.4, and 5.2 for normal hemoglobin, beta thalassemia trait, and Hb E trait.

## 3. Discussion

Interference with Hb A1c measurement by hemoglobin traits has been described previously [2, 5–10]. A detailed list of interference by hemoglobin variants as well as method used has been listed (http://www.ngsp.org/). In this study, we did not find difference between Hb A1c levels in patients with hemoglobin traits and normal hemoglobin. This implies that Hb E and beta thalassemia trait do not interfere with A1c measurements by HPLC on D-10 analyzer using the Hemoglobin A1c/A2/F BIO-RAD D-10 dual program. Patients with normal hemoglobin showed significant positive correlation between age and Hb A1c levels. This could be due to the number of patients with Hb E trait (48) and beta thalassemia trait (63) compared to normal hemoglobin (186) in this study. In order to investigate further we conducted a Mann-Whitney *U* test between A1c levels from patients with normal hemoglobin and those with Hb E trait and beta thalassemia trait. Hb A1c results for patients with HbE or beta thalassemia trait are not significantly different from those of normal subjects. In conclusion, Hb A1c measurement by HPLC using the extended program of D-10 analyzer is reliable in hemoglobin traits studied. However, in compound heterozygous and homozygous states, other methods for detection of Hb A1c have to be used as there is no Hb A1c peak due to absence of Hb A. According to the manufacturer Hb C, Hb D, Hb E and Hb S trait do not interfere with Hb A1c levels and value obtained by the D-10 dual extended program is similar to the boronate affinity method. Hb A1c level from patients with normal hemoglobin is not different from patients with hemoglobin traits. We also found that frequency of patients with diabetes did not differ in patients with hemoglobinopathy/thalassemia traits when compared to the normal population (10.5 and 9.8, resp.).

## Figures and Tables

**Table 1:** Median of various hemoglobin fractions separated by chromatography.

Chromatogram	Numbers	Age in years (median)	Hb A1c%	Hb A%	Abnormal Hb/elevated Hb A2%
Normal	186	32	5.3	85.8	Nil

Beta thalassemia trait	63	32	5.4	82.4	5.3

Hb E trait	48	33.5	5.2	61.0	27.3
